# The complete genome sequence of a new fowl adenovirus (Fadv-4) strain from a recent outbreak in a chicken farm in Iran

**DOI:** 10.1128/mra.00057-24

**Published:** 2024-03-22

**Authors:** Aliakbar khabiri, Arash Ghalyanchilangeroudi, Phuong Thi Kim Doan, Mohammadreza Pakbaz, Bahareh Pakbaten, Farhid Hemmatzadeh

**Affiliations:** 1 School of Animal and Veterinary Sciences, The University of Adelaide, Adelaide, Australia; 2 Department of Microbiology and Immunology, Faculty of Veterinary Medicine, University of Tehran, Tehran, Iran; 3 Pasouk Vaccine Production Company, Tehran, Iran; 4 The Davies Research Centre, School of Animal and Veterinary Sciences, The University of Adelaide, Adelaide, Australia; DOE Joint Genome Institute, Berkeley, California, USA

**Keywords:** FAdv-C, FAdv-4, hydropericardium-hepatitis syndrome (HHS)

## Abstract

The whole genomic sequence of fowl adenovirus C (FAdV-4) strain FAdV-4/Pasouk, isolated from chickens with hepatitis-hydropericardium syndrome (HHS) from an outbreak in Iran, has been deposited in GenBank under accession number ON652872. Notably, this FAdV-4 isolate exhibited significant genetic similarities to contemporary isolates originating from China, indicating a shared ancestry.

## ANNOUNCEMENT

Fowl *Adenoviruses* (FAdV), part of the *Aviadenovirus* genus in the Adenoviridae family, are classified into 12 serotypes, denoted as FAdV-1 to FAdV-8a and FAdV-8b to FAdV-11, and further organized into five species, namely FAdV-A to FAdV-E (1, 2). This viral group has emerged as a significant global threat to the poultry industry, exerting notable influences on growth and productivity (3, 4). Its association with severe diseases, including hepatitis-hydropericardium syndrome (HHS) and inclusion body hepatitis (IBH), underscores its impact (5, 6). Notably, FAdV, particularly subtype FAdV-1, has been linked to a distinctive syndrome recognized as gizzard erosion in various countries (7, 8). Presently, FAdV-4 prevails in Iran and spreads in different poultry sectors in the Middle East (9, 10). The whole-genome sequence of the Fadv-4 strain presented in this study has been deposited in GenBank under the accession number ON652872 and it serves as a valuable resource for future analysis of latest FadV strains circulating in Iran.

**Fig 1:**
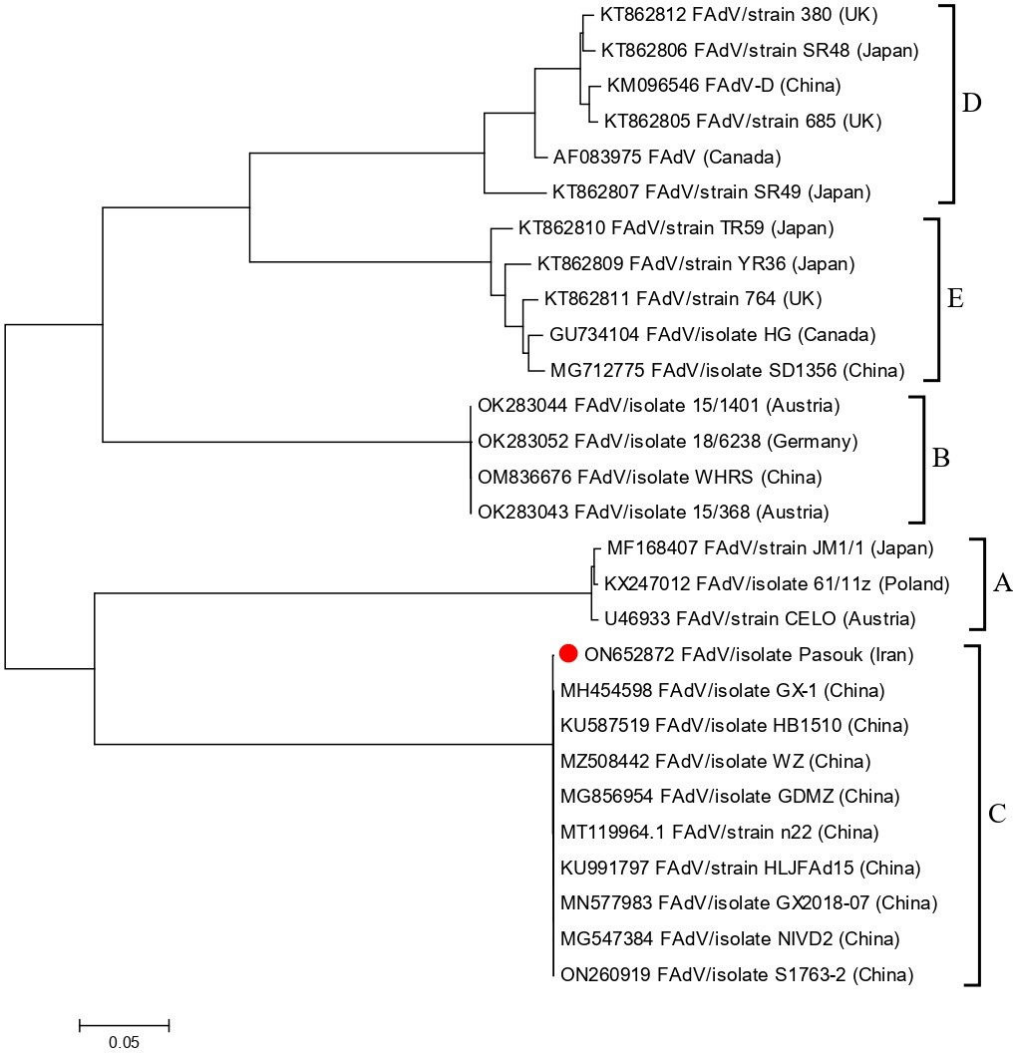
Phylogenetic tree based on the complete genome sequences of FAdV-4 Pasuk isolated. The multiple alignment with available sequences from GenBank was conducted using the ClustalW program (https://www.genome.jp/tools-bin/clustalw) and the tree was constructed by the ML method with 1,000 bootstrap replications.

The source of the isolate was liver tissue that was collected from a chicken farm in Tehran province in February 2021, with the clinical symptoms of HHS including hepatitis, hydropericardium, gizzard erosion, and high mortality. The liver sample was homogenized and inoculated into 9-day-old specific-pathogen-free (SPF) chicken eggs. For further purification, the isolated virus was inoculated into a hepatocellular carcinoma epithelial cell line (LMH) sourced from American Type Culture Collection (ATCC) with the code number CRL-2117. The LMH cells that showed cytopathic effects (CPE) were tested in a specific PCR for FAdVs (11). The extracted DNA (DNeasy Blood & Tissue Kits, Qiagene) from PCR-positive viral samples were subjected to whole-genome sequencing (Macrogen Oceania, NSW, Australia). The Illumina MiSeq platform v3, using 2 × 151-nucleotide (nt) paired-end [PE] mode, was used to sequence the DNA libraries prepared with the Nextera XT DNA Library Prep Kit Reference Guide (15031942 v03). On the Galaxy platform (12), Trimmomatic was employed to remove adapters and low-quality reads, while Velvet Optimiser was utilized for *de novo* assembly, resulting in a total of 7,009,767 reads with an average coverage depth of 160×. All tools were run with default parameters. The sequence was edited using Integrative Genetic Viewer (IGV) version 2.3.79 (13). The obtained Contigs was 38,694 nucleotides long with a G + C content of 54.85%. A nucleotide blast comparison with full Adenovirus genomes available in GenBank (Nov 2023) revealed 100% query cover and 99.94% identity with Fowl Aviadenovirus C (GenBank accession number MT119964) isolated in China. In all, 63 open reading frames (ORFs) were predicted within the genome using the ORF finder tool (https://www.ncbi.nlm.nih.gov/orffinder/). The genome was utilized for phylogenetic analysis due to its variability and ability to differentiate between serotypes and strains. The analysis was carried out using Mega X software (14). Classification of Aviadenovirus genotypes based on whole-genome phylogenetic trees confirmed that the virus under study belongs to the FAdV-4 genotype (Fig. 1).

## Data Availability

Raw MiSeq data have been deposited in the NCBI Sequence Read Archive (SRA) under accession number SRR26907426. The complete genome sequence of FAdV-4 is accessible in GenBank under accession number ON652872.

## References

[1] Chen L, Yin L, Zhou Q, Peng P, Du Y, Liu L, Zhang Y, Xue C, Cao Y. 2019. Epidemiological investigation of fowl adenovirus infections in poultry in China during 2015–2018. BMC Vet Res 15:271. doi:10.1186/s12917-019-1969-7 31370846 PMC6676587

[2] Kaján GL, Kecskeméti S, Harrach B, Benkő M. 2013. Molecular typing of fowl adenoviruses, isolated in Hungary recently, reveals high diversity. Vet Microbiol 167:357–363. doi:10.1016/j.vetmic.2013.09.025 24139719

[3] Hoerr FJ. 2010. Clinical aspects of immunosuppression in poultry. Avian Dis 54:2–15. doi:10.1637/8909-043009-Review.1 20408392

[4] Legnardi M, Tucciarone CM. 2020. Infectious bronchitis virus evolution, diagnosis and control. Vet Sci 7:79. doi:10.3390/vetsci7020079 32580381 PMC7356646

[5] El-Shall NA, El-Hamid HSA, Elkady MF, Ellakany HF, Elbestawy AR, Gado AR, Geneedy AM, Hasan ME, Jaremko M, Selim S, El-Tarabily KA, El-Hack MEA. 2022. Epidemiology, pathology, prevention, and control strategies of inclusion body hepatitis and hepatitis-hydropericardium syndrome in poultry: a comprehensive review. Front Vet Sci 9:963199. doi:10.3389/fvets.2022.963199 36304412 PMC9592805

[6] De Luca C, Schachner A, Mitra T, Heidl S, Liebhart D, Hess M. 2020. Fowl adenovirus (FAdV) fiber-based vaccine against inclusion body hepatitis (IBH) provides type-specific protection guided by humoral immunity and regulation of B and T cell response. Vet Res 51:143. doi:10.1186/s13567-020-00869-8 33267862 PMC7709361

[7] Şahindokuyucu İ, Çöven F, Kılıç H, Yılmaz Ö, Kars M, Yazıcıoğlu Ö, Ertunç E, Yazıcı Z. 2020. First report of fowl aviadenovirus serotypes FAdV-8B and FAdV-11 associated with inclusion body hepatitis in commercial broiler and broiler-breeder flocks in Turkey. Arch Virol 165:43–51. doi:10.1007/s00705-019-04449-w 31676996

[8] Hess M. 2013. Aviadenovirus infections, p 290–300. In Diseases of poultry

[9] Bayraktar E, Aydin O, Tali HE, Yilmaz SG, Yilmaz A, Turan N, Bamac OE, Ozturk A, Kelleci M, Sadeyen J-R, Chang P, Iqbal M, Yilmaz H. 2023 Molecular characterisation of fowl adenovirus associated with hydropericardium hepatitis syndrome in broiler and layer breeders in Azerbaijan. Biology and Life Sciences. doi:10.20944/preprints202304.0236.v1 PMC11157802 38849870

[10] Shaib H, Ramadan N, Mahmoud G, Nassif G. 2017 Outbreak of inclusion body hepatitis causing adenovirus in lebanese broiler flocks. EC Microbiol 13:92–101.

[11] Dou Y, Zheng X, Chen H, Yu X, Zhang Y, Zhang M. 2017 Isolation and identification of the fowl adenovirus serotype 4 from chicken. J Vet Sci 37:1036–1040.

[12] Afgan E, Baker D, Batut B, van den Beek M, Bouvier D, Cech M, Chilton J, Clements D, Coraor N, Grüning BA, Guerler A, Hillman-Jackson J, Hiltemann S, Jalili V, Rasche H, Soranzo N, Goecks J, Taylor J, Nekrutenko A, Blankenberg D. 2018. The Galaxy platform for accessible, reproducible and collaborative biomedical analyses: 2018 update. Nucleic Acids Res 46:W537–W544. doi:10.1093/nar/gky379 29790989 PMC6030816

[13] Robinson JT, Thorvaldsdóttir H, Winckler W, Guttman M, Lander ES, Getz G, Mesirov JP. 2011. Integrative genomics viewer. Nat Biotechnol 29:24–26. doi:10.1038/nbt.1754 21221095 PMC3346182

[14] Stecher G, Tamura K, Kumar S. 2020. Molecular evolutionary genetics analysis (MEGA) for macOS. Mol Biol Evol 37:1237–1239. doi:10.1093/molbev/msz312 31904846 PMC7086165

